# Supplementary material for: Lineage does not regulate the sensory synaptic input of projection neurons in the mouse olfactory bulb
Source: eLife. 2019 Aug 27;8:e46675. doi: 10.7554/eLife.46675 (PMC6744224; doi:10.7554/eLife.46675)
Supplement: Supplementary file 1. [file elife-46675-supp1.docx]

|  | **MOB** | **AOB** |
| --- | --- | --- |
| 3 OBs | RFP | 2-3 cell RFP |
| 1 OB | YFP+RFP | 3 cells YFP |
| 1 OB | YFP | 1 cell RFP |
| 1 OB | CFP | 1 cell RFP |
| 3 OBs | X | 1-2 cell RFP |
| 1 OB | X | 1 cell YFP |
| 1 OB | CFP | X |
| 1 OB | YFP | X |
| 4 OBs | RFP | X |
| 2 OBs | RFP+YFP | X |
| 10 OBs | x | x |

**Supplementary File 1**
