# Supplementary material for: Lineage does not regulate the sensory synaptic input of projection neurons in the mouse olfactory bulb
Source: eLife. 2019 Aug 27;8:e46675. doi: 10.7554/eLife.46675 (PMC6744224; doi:10.7554/eLife.46675)
Supplement: Transparent reporting form [file elife-46675-transrepform.pdf]

eLIFE
1st Floor
24 Hills Road
Cambridge CB2 1JP, UK
P 01223 855340
W elifesciences.org
T @elife

eLife's transparent reporting form

We encourage authors to provide detailed information *within their submission* to facilitate the interpretation and replication of experiments. Authors can upload supporting documentation to indicate the use of appropriate reporting guidelines for health-related research (see EQUATOR Network), life science research (see the BioSharing Information Resource), or the ARRIVE guidelines for reporting work involving animal research. Where applicable, authors should refer to any relevant reporting standards documents in this form.

If you have any questions, please consult our Journal Policies and/or contact us: editorial@elifesciences.org.

Sample-size estimation

- You should state whether an appropriate sample size was computed when the study was being designed
- You should state the statistical method of sample size computation and any required assumptions
- If no explicit power analysis was used, you should describe how you decided what sample (replicate) size (number) to use

Please outline where this information can be found within the submission (e.g., sections or figure legends), or explain why this information doesn't apply to your submission:

In the results and materials and methods section we state how the number of cells per clone, and the distribution of sister cells in each clone was sufficiently similar between clones to assume that our analysis identified representative clones. We also indicate the possibility that they may exist rare (non-representative) clones, that we failed to identify in our study, and that these clones may contain cells where sister neurons receive the same synaptic input.
In the submission we indicate in the main "results" section of the ms. that although it was not the original objective of our study, the reviewers requested information regarding clonal analysis in the accessory olfactory bulb (AOB). At their request, we briefly mentioned this information about the AOB in the ms., but because the number of clones with cells labeled in the AOB is small, in the results section we clearly state that the small sample does not allow one to draw any conclusions regarding the AOB.

Replicates

- You should report how often each experiment was performed
- You should include a definition of biological versus technical replication
- The data obtained should be provided and sufficient information should be provided to indicate the number of independent biological and/or technical replicates
- If you encountered any outliers, you should describe how these were handled
- Criteria for exclusion/inclusion of data should be clearly stated
- High-throughput sequence data should be uploaded before submission, with a private link for reviewers provided (these are available from both GEO and ArrayExpress)

Please outline where this information can be found within the submission (e.g., sections or figure legends), or explain why this information doesn't apply to your submission:

eLife Sciences Publications, Ltd is a limited liability non-profit non-stock corporation incorporated in the State of Delaware, USA, with company number 5030732, and is registered in the UK with company number FC030576 and branch number BR015634 at the address 1st Floor, 24 Hills Road, Cambridge CB2 1JP | August 2014

This information is clearly stated in the results section, in the figure legends and in the material and methods section.

Statistical reporting

- Statistical analysis methods should be described and justified
- Raw data should be presented in figures whenever informative to do so (typically when N per group is less than 10)
- For each experiment, you should identify the statistical tests used, exact values of N, definitions of center, methods of multiple test correction, and dispersion and precision measures (e.g., mean, median, SD, SEM, confidence intervals; and, for the major substantive results, a measure of effect size (e.g., Pearson's r, Cohen's d)
- Report exact p-values wherever possible alongside the summary statistics and 95% confidence intervals. These should be reported for all key questions and not only when the p-value is less than 0.05.

Please outline where this information can be found within the submission (e.g., sections or figure legends), or explain why this information doesn't apply to your submission:

We used two-way ANOVA to compare the NNDs of real OB clones between them (figure legend in Figure 3) and the NNDs of real OB clones with simulate random OB clones (main text line 130 and figure legend in Figure 3).

We also compared the NNDs of real neocortex clones between them (legend in Figure 3) and the NNDs of real neocortex clones with simulate random neocortex clones (main text line 130 and figure legend in Figure 3).

We used unpaired two-tailed t-test to compared the cell distribution in the dorsal and ventral domains for OB clones when progenitor cells were labeled at E10.5 (figure legend in Figure 3-figure supplement 1) and E12.5 (Figure 3-figure supplement 4).

(For large datasets, or papers with a very large number of statistical tests, you may upload a single table file with tests, Ns, etc., with reference to sections in the manuscript.)

Group allocation

- Indicate how samples were allocated into experimental groups (in the case of clinical studies, please specify allocation to treatment method); if randomization was used, please also state if restricted randomization was applied
- Indicate if masking was used during group allocation, data collection and/or data analysis

Please outline where this information can be found within the submission (e.g., sections or figure legends), or explain why this information doesn't apply to your submission:

Group allocation does not apply to our study because we were not trying to compare between groups. The objective of the study was to analyze whether sister cells in a clone in the olfactory bulb received the same synaptic input or not. This information is stated in the results section, in the figure legends, and in the materials and methods.

Additional data files ("source data")

- We encourage you to upload relevant additional data files, such as numerical data that are represented as a graph in a figure, or as a summary table
- Where provided, these should be in the most useful format, and they can be uploaded as “Source data” files linked to a main figure or table
- Include model definition files including the full list of parameters used
- Include code used for data analysis (e.g., R, MatLab)
- Avoid stating that data files are “available upon request”

Please indicate the figures or tables for which source data files have been provided:

We included all the datasets obtained from our near neighbor distance (NND) in the olfactory bulb and neocortex at E10.5, as well as the dataset obtained from the simulate random NNDs from OB and neocortex (E10.5) shown in **Figure 3 - source data 1.**

We included all the datasets obtained from our near neighbor distance (NND) in the olfactory bulb and neocortex at E12.5 shown in **Figure 3 - source data 2.**

eLife Sciences Publications, Ltd is a limited liability non-profit non-stock corporation incorporated in the State of Delaware, USA, with company number 5030732, and is registered in the UK with company number FC030576 and branch number BR015634 at the address 1st Floor, 24 Hills Road, Cambridge CB2 1JP | August 2014
